# Comparison of pathway analysis and constraint-based methods for cell factory design

**DOI:** 10.1186/s12859-019-2934-y

**Published:** 2019-06-20

**Authors:** Vítor Vieira, Paulo Maia, Miguel Rocha, Isabel Rocha

**Affiliations:** 1 Centro de Engenharia Biológica, Universidade do Minho, Braga, Portugal; 2 SilicoLife Lda, Braga, Portugal; 3 Instituto de Tecnologia Química e Biológica António Xavier, Universidade Nova de Lisboa (ITQB-NOVA), Oeiras, Portugal

**Keywords:** Genome-scale metabolic models, Computational strain design, Metabolic pathway analysis, Evolutionary algorithms, Minimal cut sets, Growth-coupled product synthesis

## Abstract

**Background:**

Computational strain optimisation methods (CSOMs) have been successfully used to exploit genome-scale metabolic models, yielding strategies useful for allowing compound overproduction in metabolic cell factories. Minimal cut sets are particularly interesting since their definition allows searching for intervention strategies that impose strong growth-coupling phenotypes, and are not subject to optimality bias when compared with simulation-based CSOMs. However, since both types of methods have different underlying principles, they also imply different ways to formulate metabolic engineering problems, posing an obstacle when comparing their outputs.

**Results:**

In this work, we perform an in-depth analysis of potential strategies that can be obtained with both methods, providing a critical comparison of performance, robustness, predicted phenotypes as well as strategy structure and size. To this end, we devised a pipeline including enumeration of strategies from evolutionary algorithms (EA) and minimal cut sets (MCS), filtering and flux analysis of predicted mutants to optimize the production of succinic acid in *Saccharomyces cerevisiae*. We additionally attempt to generalize problem formulations for MCS enumeration within the context of growth-coupled product synthesis. Strategies from evolutionary algorithms show the best compromise between acceptable growth rates and compound overproduction. However, constrained MCSs lead to a larger variety of phenotypes with several degrees of growth-coupling with production flux. The latter have proven useful in revealing the importance, in silico, of the gamma-aminobutyric acid shunt and manipulation of cofactor pools in growth-coupled designs for succinate production, mechanisms which have also been touted as potentially useful for metabolic engineering.

**Conclusions:**

The two main groups of CSOMs are valuable for finding growth-coupled mutants. Despite the limitations in maximum growth rates and large strategy sizes, MCSs help uncover novel mechanisms for compound overproduction and thus, analyzing outputs from both methods provides a richer overview on strategies that can be potentially carried over in vivo.

**Electronic supplementary material:**

The online version of this article (10.1186/s12859-019-2934-y) contains supplementary material, which is available to authorized users.

## Background

Genome-scale metabolic models (GSMM) are well proven tools for the *in-silico* analysis of the metabolism of living organisms. Indeed, the wide availability of whole-genome sequencing and annotation tools have enabled the reconstruction of a multitude of metabolic networks for various organisms [1]. Constraint-based (CB) modelling approaches allow the usage of GSMMs for simulation, analysis and strain optimization purposes which, despite the increasing scale of these models, have proven useful for a variety of studies. Phenotype prediction methods such as Flux Balance Analysis (FBA) [2] and its variants for mutant phenotypes [3–5], as well as Elementary Modes Analysis (EMA) [6] are capable of providing valuable insights on cell metabolism.

Designing optimized microbial strains for compound overproduction, however, is achieved through computational strain optimization methods (CSOMs), providing a rational approach for finding intervention strategies, as opposed to trial-and-error experiments [7, 8]. The purpose of most available CSOMs in the metabolic engineering (ME) context is to find sets of reactions that, when modified, force the cell to couple fluxes involved with the production of the desired compound with those required for cell growth. Growth-coupled phenotypes can be classified as weak, when compound production is only forced above a certain growth rate threshold, or strong when the fluxes driving the production of the desired compound are essential for cell growth [9].

CSOMs can be branched in two main categories: simulation-based (SB) and EMA-based methods. For simplification purposes we group bi-level mixed integer programming (MIP) and metaheuristic methods as SB methods, due to their similarities in evaluating candidate strategies, although with different methods for generating them (as recently reviewed by Maia and co-workers [8] and Machado et al. [10]).

SB CSOMs mostly derive from the bi-level framework first presented by Burgard and colleagues in the OptKnock approach [11], defining a strategy where an optimization layer is subject to the constraints posed by CB models. Several variations of this MIP problem have also been proposed to find more robust strategies and integrate omics data in the search [12, 13]. On the other hand, the OptGene approach later presented by Patil et al. [14], first introduced the usage of genetic algorithms for the optimization layer, which effectively detaches it from the simulation allowing for more flexible objective definitions and reducing the computational cost. Several improvements were subsequently published, including alternative evolutionary algorithms [15] and multiple objective functions [16]. Until recently, this type of CSOM comprised the only computationally feasible choice for strain optimization in GSMMs.

EMA-based CSOMs, on the other hand, search strategies for the desired ME goals throughout the entire solution space, resulting in predicted phenotypes that are not reliant on optimality assumptions. This implies an increase in computational demand, which severely hinders the scalability of most CSOMs of this type [8]. A prominent example is the concept of minimal cut sets (MCSs) [17], the smallest intervention targets that block a certain phenotype.

MCS enumeration algorithms were mostly reliant upon complete elementary mode (EM) enumeration, rendering it an infeasible task for models of greater scale and complexity. Several methods have been developed to tackle the computational limitations of EMA-based CSOMs by allowing partial enumeration of EMs through the use of sampling approaches [18], evolutionary algorithms [19], as well as other methods [20, 21]. The *k*-shortest EM method [21] is a relevant example of this, allowing enumeration of the *k* smallest EMs using a MILP (mixed-integer linear programming) approach.

The MCSEnumerator approach recently proposed by von Kamp et al. [22] successfully employs K-shortest EM enumeration in a dual linear problem through which EMs can be mapped to MCSs on the original network, as demonstrated by Ballerstein and colleagues [23]. The tool’s feasibility for GSMMs has been demonstrated with the enumeration of synthetic lethals and knockout strategies for production of various compounds using a model for *Escherichia coli* [22]. Erdrich et al. have also explored this tool as a means to find design strategies for biofuel production in cyanobacteria [24] and a recent in vivo study has also confirmed its importance as a rational strain design tool for itaconate production in *Escherichia coli*, albeit using a smaller scale model [25].

Despite the existence of some studies highlighting the importance of EMA-based CSOMs to solve ME problems [24, 25], there is a gap regarding the analysis of these methods’ outputs. Von Kamp and Klamt have recently assessed the feasibility of growth-coupled product synthesis in various organisms [26]. However, the application of these methods for strain optimization is still limited and few studies have discussed the biological implications of strategies obtained using these approaches. Recent developments by Harder et al. use an iterative rational strain design approach with successful in vivo outcomes, but do not apply MCSs directly as design strategies, relying instead on continuously applying partial MCSs and updating the metabolic model’s environmental conditions for subsequent enumerations [25].

The comparison between EMA and SB CSOM derived strategies is also often overlooked. Most SB CSOMs are subject to optimality bias since they require an objective function which is often misleading and hard to define in certain organisms or metabolic systems. EMA-based CSOMs can now provide a suitable alternative to obtain highly robust design strategies with a low number of modifications, but there is a general lack of information regarding how well these perform against state-of-the-art SB CSOMs and whether their strategies produce better in vivo candidates.

The purpose of this work is to employ EMA-based and SB CSOMs in strain design applications using a case study involving the identification of knockout strategies for the production of succinic acid with *Saccharomyces cerevisiae*. To assess the feasibility of these methods, outputs from both categories are compared and different formulations of both approaches are also analysed. The aim is to bridge the gap between EMA and SB CSOMs by assessing performance metrics for each set of strategies and understanding the advantages and limitations of each method regarding their usage for ME tasks with different production and growth demands.

We present a pipeline including strain optimization, filtering and analysis of design strategies based on previous developments and packaged it as part of the Metabolic Engineering Workbench (MEW),^1^ an open-source *Java* library developed in-house. Two additional problem formulations are also presented to allow greater flexibility of growth-coupled design strategies based on MCSs. A graphical user interface for the MCS enumeration algorithm was also made available as a plugin for the *OptFlux* metabolic engineering platform [27].

## Results

**Fig. 1:**
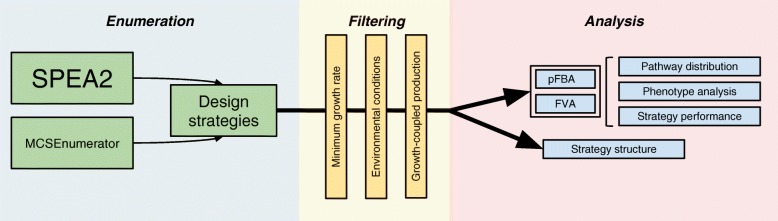
Brief overview of the pipeline employed in this work. Optimization is performed using two strain design algorithms that produce reaction deletion strategies. These are filtered according to several criteria so they are compliant with the defined environmental conditions, minimum growth rate and production demands. The selected strategies are then subjected to various analysis methods

The methodological pipeline used for this work can be divided in three key steps as illustrated in Fig. 1. These steps are detailed in the methods sections.

The primary goal with our workflow is to explore growth-coupled phenotypes through design strategies obtained with EMA-based and SB CSOMs. Five different strain design strategies, described in more detail in the Methods section, were obtained with SB CSOMs (using the evolutionary algorithm SPEA2) and EMA-based CSOMs (with MCSEnumerator) as part of our experimental setup.

Using SPEA2, biomass-product coupling maximization (*EAw*) and product minimum maximization (*EAm*) were tested, allowing different trade-offs between biomass and target compound production. *EAw* includes maximum cell growth and production flux at maximum cell growth as two separate objectives, while *EAm* uses the maximum cell growth rate and minimum product flux at maximum growth as objectives.

For MCSEnumerator, three strategies (*MCSe*, *MCSf* and *MCSw*) cover previously used and novel approaches that are more or less strict regarding robustness of the solutions by different definitions of the undesired spaces. In *MCSe* the undesired space contains low product yield flux vectors with a maximum substrate uptake rate and maintenance ATP rate above a defined value. In *MCSf*, low product yield phenotypes are blocked, assuming a fixed substrate uptake rate. In *MCSw*, low product flux phenotypes are blocked only when biomass fluxes are above a fraction of the maximum growth rate, aiming to reach strategies with less strict demands regarding product synthesis and its coupling with growth.

The purpose is to compare the five chosen strategies, considering a ME case study with the production of succinic acid with *Saccharomyces cerevisiae* as an objective. All analyses consider glucose as a carbon source with an uptake flux of 1.15 mmol.gDW^− 1^.h^− 1^ and aerobic conditions. For validation purposes, we also test different MCS formulations with previously determined Escherichia coli strategies also derived from MCSs. The results from this case study can be found as part of the Additional file 1.

### Strategy performance

**Fig. 2:**
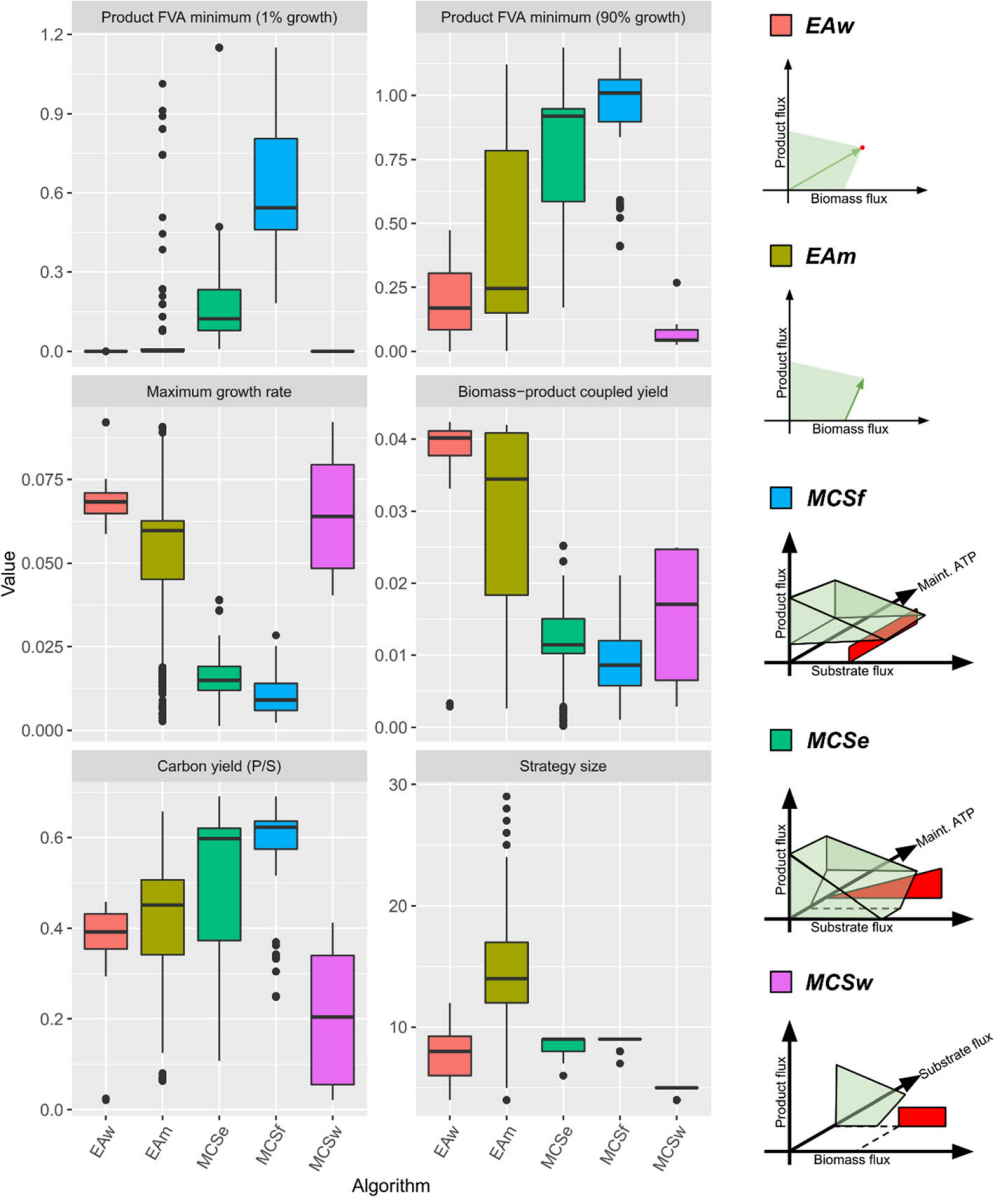
Left: Overview of various performance and robustness metrics for the solution sets featured by the analysis of succinate producing strategies. Displayed metrics: Production robustness (at 1 and 90% of maximum biomass), maximum growth rate, biomass-product coupled yield and product/substrate carbon yield. Right: Representation of the formulations featured in this case study. Colours match the ones used in the left part of the image. The undesired flux space is represented in red (only for cMCS enumeration problems), while the expected phenotype is represented in light green. For evolutionary algorithms, a green arrow represents the objective function

First, we compare the predicted performance of strategies from all approaches. The results for this analysis are highlighted on Fig. 2.

#### Production robustness

Strategies from the *MCSe* and *MCSf* sets provide fully robust production phenotypes with forced product synthesis even at very low growth rates (strongly-coupled). SPEA2 and *MCSw* strategies lead to lower product flux values and only guarantee product synthesis at higher growth rates. It is worth noting that a considerable amount of *MCSw* strategies do not allow growth-coupling at all, or do not meet our demand for production at 90% of the maximum mutant growth rate. This explains the need for a filtering step prior to our analysis.

*EAm* strategies lead to moderately robust phenotypes with higher product rates across different cell growth thresholds, with some of these strategies leading to strongly growth-coupled production phenotypes. These results are expected, considering the constraints imposed upon the various formulations.

The maintenance ATP constraint imposed on the *MCSe* formulation seems to negatively affect production robustness and rates, as this set underperforms the *MCSf* regarding these properties.

#### Cell growth and productivity

*MCSf* and *MCSe* strategies appear to be the worst performing strategies when considering cell growth. BPCY values for these sets are similar and considerably lower than for EA solutions. Despite the increased product flux values, strongly coupled MCSs (*MCSe* and *MCSf*) lead to much lower growth rates, which negatively impacts BPCY.

Conversely, the *MCSw* solution set, with lower production rates and robustness, leads to similar BPCY values since the maximum cell growth is much higher. *EAw* solutions reach the highest average BPCY figures, surpassing the *EAm* set, which, despite the increased product flux values, leads to lower maximum growth rates.

Regarding carbon yields, all strategy sets predict maximum glucose uptake, leading to carbon yields that are directly dependent upon the product flux. Thus, *MCSe* and *MCSf* solutions lead to higher carbon yields, followed by the *EAm* and *EAw* set. *MCSw* strategies have noticeably lower production rates, also leading to lower carbon yields.

The MCSe set appears to lead to slightly higher maximum growth rates and BPCY than the MCSf set. Maximum carbon yield, however, is lower (due to lower product rates).

The size of the analysed strategies is not homogeneous across all groups. MCS derived formulations are guaranteed to deliver the smallest solutions for the specified problem. SPEA2, being a heuristic approach, may lead to bigger solutions. Despite the large range of solution sizes for formulations based on this algorithm, EA-derived strategies with the highest BPCY values contain less than 10 knockouts. Additionally, the smallest strategies for succinate production are found in these solution sets (less than 5 knockouts), even though the average solution size across the entire sets is higher.

MCS derived strategies have low ranges as the number of solutions increases heavily with the proposed size, which imposes a computational limit. *MCSw* solutions were limited to sizes 3 through 5, while *MCSe* and *MCSf* were achieved with sizes up to 9 deletions. Productivity appears to increase with solution size in this particular case. However, the aforementioned changes in robustness and productivity metrics can be verified even when comparing similar solution sizes.

### Phenotype analysis

**Fig. 3:**
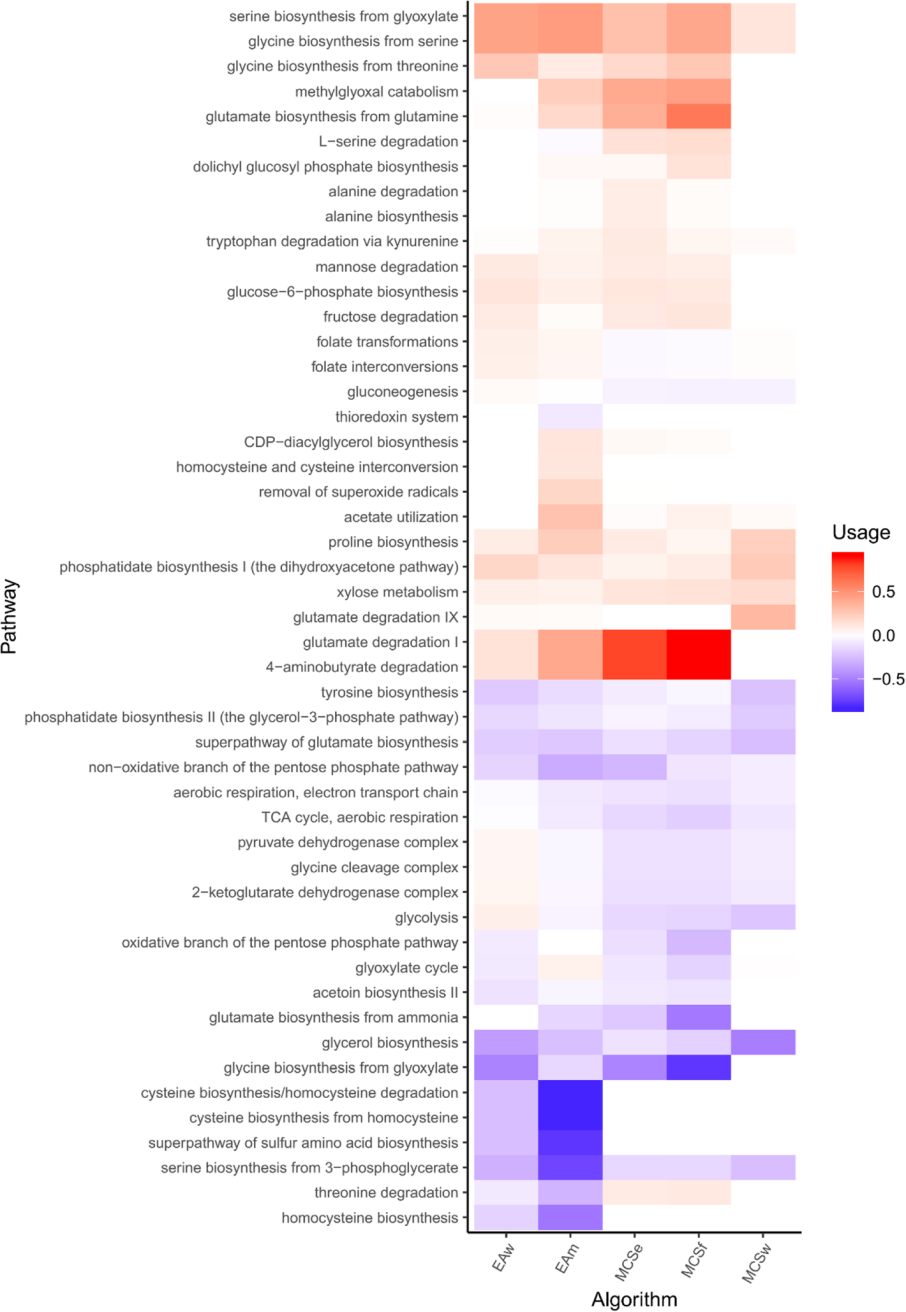
Overview of the usage of various pathways for the solutions obtained by each of the different design strategies for succinate production. The values represent the difference in the proportion of active reactions when compared with the wild-type simulated with parsimonious FBA. Pathway nomenclature was obtained on BioCyc and matched with KEGG identifiers assigned to reactions on the GSMM

The main results of the pathway distribution approach used in this work are depicted on Fig. 3, showing the activity of specific pathways towards the goal of achieving different growth-coupled phenotypes. Average flux values throughout the strategy sets were compared and merged into their respective pathways to illustrate this.

Across the solutions from all strategies, there is a decreased usage of the oxidative branch of the pentose phosphate pathway (PPP), as compared to the wild-type. This correlates with the knockouts themselves on the pathway, found in *EAw* and both MCS solution sets. Flux on the non-oxidative branch is also generally decreased, although some reactions leading up to the amino-acid biosynthetic pathways remain active, which can be observed on Fig. 3. This can be seen through the increased usage of alanine, serine, glycine and glutamate biosynthetic pathways in strongly-coupled strategies and the *EAm* set.

The tricarboxylic acid (TCA) cycle is also disrupted in most of the proposed designs, leading to less active reactions in this pathway. This also leads to a lower activity in the electron transfer chain. Interestingly, the solutions from the *EAw* set, leading to higher growth rates, maintain flux in TCA cycle reactions, whereas the solutions from the remaining strategies do not. This could be explained by the high usage of fumarate reductase and the glyoxylate shunt (albeit with smaller flux) which is the primary mechanism found for succinate production in *EAw* solutions, in contrast with 4-aminobutyrate (GABA) degradation pathways used in strongly-coupled solutions.

The GABA degradation pathway plays a key role in the solutions obtained from strongly-coupled strategies. This pathway, otherwise known as the GABA shunt, leads to succinate production through degradation of GABA in the cytosol and serves as the main production mechanism in all *MCSf* and *MCSe* solutions and in a smaller proportion in *EAm*. Increased usage of this pathway seems to directly correlate with higher production robustness. Similarly, usage of the methylglyoxal catabolic pathway is also increased in solutions from *MCSf*, *MCSe* and *EAm* strategies. Through further analysis of the admissible flux ranges using FVA we were also able to find that GABA shunt fluxes become essential for the feasibility of most *MCSf* and *MCSe* solutions, which also shows that growth and production are coupled to this pathway.

It is worth noting that the strongly growth-coupled phenotypes we found were dependent on the imbalance of at least one cofactor. This finding is supported by the fact that the fixed maintenance ATP demand is essential to impose strong coupling – only weak coupling is achieved if the maintenance ATP pseudo-reaction bounds are lower. Additionally, in many *MCSf* solutions, the NADPH/NADP balance is disrupted through deletions that force glucose uptake through NADPH dependent pathways or flux through certain pathways dependent on these cofactors, such as folate interconversions, or methylglyoxal metabolism. Flux through the GABA shunt is then required to maintain a steady-state. These phenomena involving cofactor pools have already been described as possible mechanisms for growth-coupled synthesis by Erdrich et al. [24] and Hädicke et al. [28], although further experimental validation is still required.

### Knockout frequency analysis

**Fig. 4:**
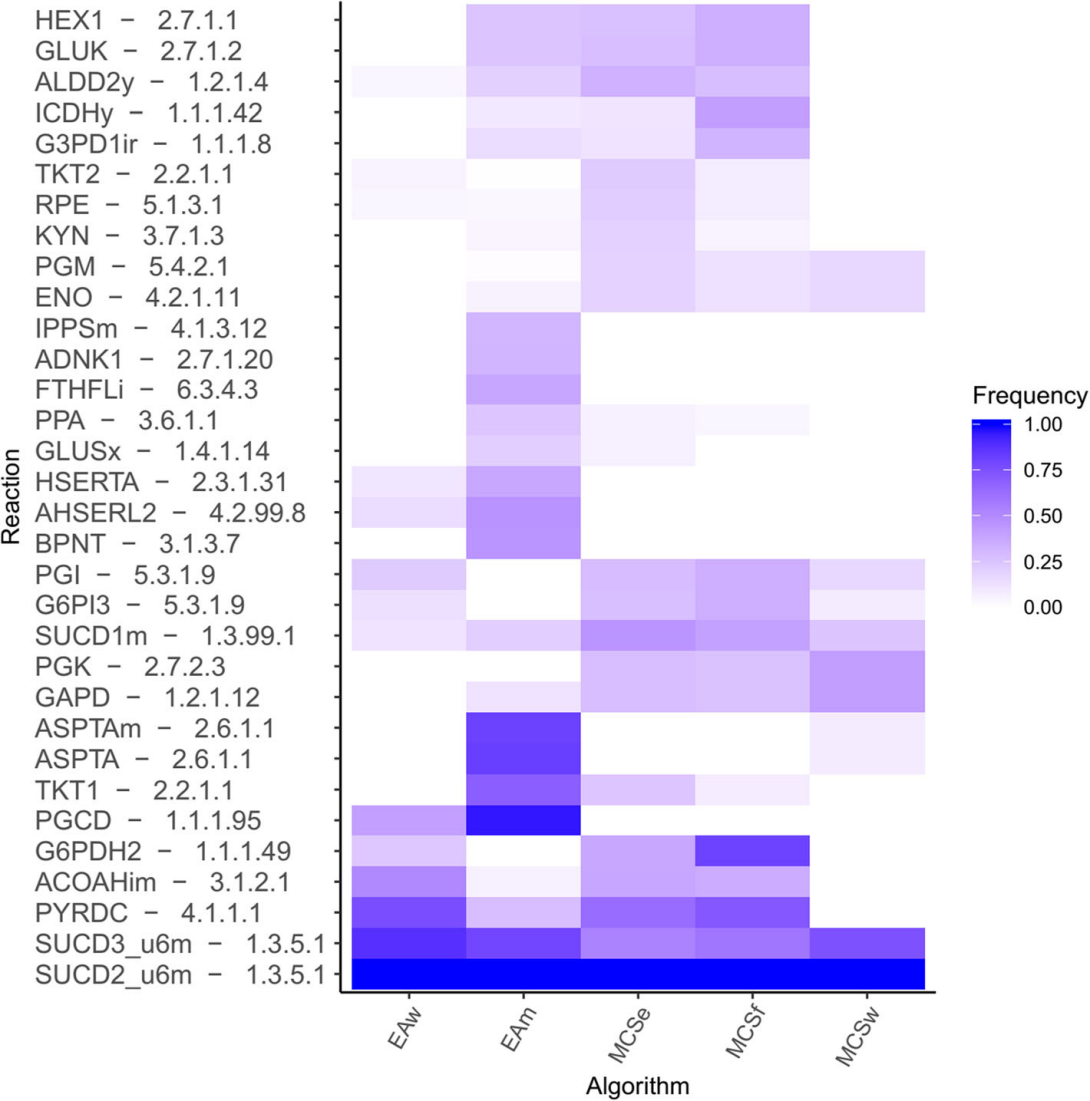
Relative frequencies of the most common knockout combinations across all solution sets from the succinate case study. Darker shades of blue represent higher knockout frequencies. Labels for each reaction include its identifier in the model as well as the Enzyme Commission number attributed to it according to the iMM904 model reconstruction used in this work

Analysis of the specific knockouts included in the obtained strategies is a key feature to determine which are the most important deletions leading to weak or strong coupling phenotypes. Figure 4 summarizes the frequency of the most common knockouts across all formulations.

A common disruption across the solutions from all strategies is the succinate dehydrogenase complex, a TCA cycle mitochondrial protein complex supplying electrons for the transfer chain and catalysing the conversion of succinate to fumarate. Subunit 2 (EC 1.3.5.1) of this complex is present in every solution, with subunits 3 and 1 being less present (ECs 1.3.5.1 and 1.3.99.1, respectively), in decreasing order of frequency. The importance of this disruption may come from the fact that this is the only available irreversible conversion of succinate to fumarate.

Glycolysis appears as a target for disruption in strategies with higher production robustness (*EAm*, *MCSe* and *MCSf*), with higher frequency of knockouts in kinases involved in glucose phosphorylation, such as hexokinase or glucokinase. The presence of PGI (EC 5.3.1.9) knockouts in most strategies may help explain the increased flux in the PPP and alternative glucose catabolic pathways.

The EA set includes many solutions involving serine biosynthesis from intermediate metabolites of glycolysis, blocking a possible branched pathway from glycolysis. MCS strategies, on the other hand, include knockouts that block glycolysis before this branching, namely PGK and GAPD (ECs 2.7.2.3 and 1.2.1.12, respectively), which force the usage of alternative pathways, such as methylglyoxal catabolism or serine biosynthesis.

Due to the decreased rates in the oxidative phosphorylation, fermentation appears as an alternative for pyruvate catabolism for many solutions. As such, succinate producing strategies with higher titres tend to include knockouts in PYRDC (EC 4.1.1.1), ALDD (EC 1.2.1.4) and ACOAH (3.1.2.1) reactions to reduce the number of viable alternative pathways that could drive carbon away from succinate production.

Knockouts in the PPP are somewhat prevalent across all strategy sets except *MCSw*. Regarding oxidative-phase knockouts, G6PDH (EC 1.1.1.49) appears in strongly-coupled MCSs with relatively high frequencies, especially in the *MCSf* set and in the *EAw* set.

Finally, in the TCA cycle, the cytosolic IDH (EC 1.1.1.42) knockout seems to be a relatively common occurrence in *MCSf* solutions, which leads to strongly coupled succinate production through forced usage of the TCA cycle producing 2-oxoglutarate in the mitochondria and then transported outside to feed the GABA shunt for cytosolic succinate to be synthesized.

### Experimental studies

Two metabolic routes for succinate overproduction from the TCA cycle have been described in the literature, respectively, the oxidative and reductive branches of the TCA cycle [29]. The oxidative route yields a theoretical maximum of 1 mol succinate/mol glucose. Metabolic engineering strategies have been tried and tested successfully for this first route [30, 31]. The reductive pathway, usually associated with anaerobic conditions, involves succinate production through carbon dioxide fixation and relies on pyruvate carboxylation into oxaloacetate from which TCA metabolites up to fumarate can be synthesized. Fumarate reductase catalyses the conversion from fumarate to succinate.

Early efforts using yeasts as succinate-overproducing microbes have focused on the enhancement of sake brewing processes with *Saccharomyces cerevisiae* [32]. This study describes, among others, a strain with a succinate dehydrogenase (SDH) gene disruption that leads to increased succinate production in aerobic conditions. A similar finding is confirmed in another study, showing that disruption of any of the SDH subunits leads to increased aerobic succinate production and that higher succinate titres could be correlated with lower SDH complex activity [33].

Additionally, it is shown that growth and substrate uptake are not significantly altered in strains with disrupted pairs (SDH1 and SDH2) [33]. The structural analysis featured in this work reveals that inactivation of at least two subunits of the SDH system (along with further knockouts) is required to allow strongly coupled production of succinate. Another yeast strain with a SDH2 knockout was experimentally tested, albeit on a different species (*Yarrowia lipolytica*), resulting in production at a minimum of 56% of the theoretical maximum [34]. The results obtained in our analysis are in accordance with experimental data regarding the SDH2 knockout which is an essential component of strongly coupled strategies.

Further studies found improved strategies for aerobic succinate production in yeast. The addition of a isocitrate dehydrogenase (IDH) knockout to the SDH1/2 pair was studied as a means of diverting citric acid cycle intermediates into the glyoxylate cycle, resulting in succinate overproduction [31]. This mutant was able to achieve a yield almost four times greater than the wild-type strain and the addition of a mitochondrial isocitrate dehydrogenase knockout (IDP) leads to even higher succinate concentrations at the cost of reduced growth rate [31]. Some strongly coupled solutions featured in this work exhibit glyoxylate cycle flux, although these have low representation within the entire solution set.

An alternative strategy for glyoxylate shunt carbon redirection was presented by Otero et al. [30], involving two knockouts, namely at the SER3/SER33 gene (catalysing the PGCD reaction in the *i*MM904 metabolic model) and at subunit 3 of the SDH complex. Carbon flux is redirected towards glyoxylate to allow serine production, with succinate as a by-product of using this metabolic route. This strategy was developed using in silico tools and then experimentally tested. Not surprisingly, some of the solutions obtained in the present work include those strategies.

There is in vivo evidence that succinate production in *Saccharomyces cerevisiae* from the GABA shunt is residual in normal fermentation conditions (with oxygen limitation) [35]. It is still unclear whether the GABA shunt is a valid alternative for succinate production in aerobic conditions, but our results make this pathway a relevant candidate for further study in aerobic conditions.

Regarding the reductive pathway, not many efforts have been implemented regarding the optimization of this route, mainly due to cofactor imbalances. However, reported strategies involve the deletion of fermentative competing pathways, as observed in some of our solutions [36].

## Discussion

In this work, we present a comparison of two relevant constraint-based strain design approaches for ME applications through in silico analysis of the resulting knockouts strategies. Two alternative formulations for MCS enumeration were tested, yielding mutants with varying growth and production demands, demonstrating the flexibility of the intervention problem framework coupled with the MCSEnumerator algorithm. The results shown in this work reinforce the importance of MCSs as a rational approach for ME applications.

When comparing EA and MCS-derived strategies, there is a clear trade-off between robustness and maximum cell growth. Growth-coupling strength increases as maximum biomass decreases, leading to strongly-coupled cMCSs with high product flux and low growth and weakly-coupled EA strategies with opposing features. Phenotypes with very high yields have been found using cMCSs, largely surpassing those from SPEA2, but cell viability is still unknown as the low cell growth rate may indicate that these strategies incur in lethal modifications in vivo.

Nevertheless, it is still possible to obtain growth-coupled phenotypes with low production robustness, using the *MCSw* formulation firstly described in this work. Strategies obtained using this method have lower predicted productivity, but overall higher growth rates. While possibly unsuitable for direct in vivo application, these strategies may serve as starting points for other strain design algorithms, upon which more knockouts can be added to achieve higher productivity.

With the *EAm* formulation, SPEA2 can also be used to obtain strongly-coupled strategies, with similar robustness to those found using MCSEnumerator. However, the latter can enumerate smaller strategies, which is advantageous for in vivo implementation.

The *MCSf* formulation presented and tested in this work allowed us to find strategies with high product flux values and robustness but lower cell growth rates overall. This helped us reveal the importance of including a coupling component (such as the maintenance ATP assumption on the *MCSe* formulation) when searching for MCS-based strategies. Indeed, when the maintenance ATP assumption is replaced with a fixed substrate constraint, other cofactor wasting mechanisms arise.

For practical purposes, it is clear that *EAw* strategies confer high BPCY at a relatively low number of knockouts to achieve that goal. However, product synthesis can only be guaranteed with growth rates close to the wild-type, which casts uncertainty on whether these strategies will have a noticeable effect in vivo.

The strong growth-coupling knockouts suggested by MCS enumeration have practical implications concerning their size, which exceeds six knockouts in the best possible case, aside from the relatively low growth rate and BPCY. However, they are promising candidates for adaptive laboratory evolution, since the target organism may acquire mutations that lead to increased growth rates while maintaining the growth-coupling with product synthesis.

Moreover, using multiple strain design approaches with varying productivity and robustness demands further helped us to find knockouts providing a beneficial compromise between maximum cell growth, production flux and yields.

The findings from the *Saccharomyces cerevisiae* case study featured in this work show that strongly growth-coupled strategies point towards increased usage of the GABA degradation pathway as production demands grows, at the cost of lower growth rates. This design strategy requires further experimental validation, despite the existence of studies acknowledging it as a potential ME target for succinate production.

This work only includes CSOMs for finding sets of reaction deletions; however, it is possible for both methods to also suggest strategies involving modification of the expression levels of metabolic genes [37], which could be addressed as a future case study.

## Conclusions

Overall, we conclude that EMA-based CSOMs are valuable tools for strain design that yield design strategies with the highest production fluxes and robustness and uncover the biochemical mechanisms that lead to these phenomena. A thorough analysis of these algorithms’ outputs serves as valuable tool for guiding rational approaches to strain optimization by highlighting relevant deletions to achieve the ME goal. Aiming for both weak and strong coupling phenotypes using these algorithms is essential towards finding a compromise between viability and productivity and identifying common targets whose modification is essential to produce a given compound. We show that it is possible to fine-tune the compromise between robustness and growth rates by choosing different algorithms or setups among those tested in this work.

## Methods

### Constraint-based modelling and analysis

In this work, we attempt to find design strategies for *Saccharomyces cerevisiae* using the *i*MM904 reconstruction presented by Mo and colleagues [38] with additional changes proposed by Pereira et al. [39] for improved predictive accuracy.

The constraint-based approach to modelling represents stoichiometry restricted by steady-state [40], through a system of linear equations complemented with inequalities representing bounds for each flux. Such a system is formulated as shown on Eq. 1, assuming **S** as a *m-*by-*n* matrix encoding the network stoichiometry, ***v*** as the flux vector, and ***l,u*** as the vectors encoding the lower and upper bounds, respectively.

1 $$ {\displaystyle \begin{array}{l}S.v=0\\ {}{l}_i\le {v}_i\le {u}_i\forall i\in \left\{1,\dots, n\right\}\\ {}l,v,u\in {\mathrm{\mathbb{R}}}^n\end{array}} $$

Typical approaches such as Flux Balance Analysis (FBA) [2] attempt to find a single flux distribution, by optimizing towards a given objective function, subject to the previously mentioned constraints.

The most common approach is to maximize cell growth, although other functions have also been proposed, mainly to address phenotype prediction in mutant cells [3, 4]. The optimization problem posed by FBA typically leads to multiple optima for the same objective, eliciting the development of alternative methods for prediction and analysis of simulated fluxes such as parsimonious FBA (pFBA) or Flux Variability Analysis (FVA).

These two methods are later used to analyse the performance of our design strategies. pFBA extends the FBA problem by constraining the optimum to that which minimizes overall flux usage (sum of absolute flux values) [5] and FVA provides theoretical maximum and minimum flux values for the model/environmental condition assuming a fraction of the maximum growth rate [41].

An alternative approach to analyse fluxes using constraint-based models is to employ convex analysis, usually with EMA methods, since the linear system of equations is a convex polyhedral cone when all reactions are irreversible [42], which is usually achieved by replacing reversible reactions with two forward and backward fluxes.

EMA is based on the concept of elementary modes (EM), defined as any flux distribution *e* that solves the system in Eq. 1 containing only irreversible reactions, such that: (1) two split reversible reactions are not simultaneously active; (2) *e* is a feasible solution, thus satisfying steady-state and thermodynamic constraints: (3) if an active reaction of *e* is removed, *e* is no longer feasible. These properties assure EMs are the smallest sets of reactions allowing valid metabolic conversions, and any steady-state flux distribution complying with thermodynamic constraints can be defined as a linear combination of the full set of EMs [6].

Minimal cut sets (MCSs) are an extension of this concept, defining sets of reactions that, when removed or blocked, disable certain selected EMs [17]. With appropriate EMs to block, it is possible to find minimal strategies to achieve or even guarantee the expression of desired phenotypes. Constrained MCSs (cMCSs) extend this concept even further to guarantee that desired phenotypes are not among those that are blocked [43].

The intervention problem framework, first presented by Hädicke et al. [43], provides a flexible approach for formulating ME problems with cMCSs. An intervention problem **I (T, D)** requires two inputs, namely:

1. Target flux space (**T**), containing undesired EMs to be blocked.
2. Desired flux space (**D**), comprising desirable EMs, which cannot be blocked.

For any problem **I**, the resulting MCSs will render the EMs in **T** invalid while also keeping EMs in **D** active. For this reason, not all MCSs for **I** can be considered cMCSs [43]. Both **T** and **D** can be defined as part of the solution space of the flux vector *v*, leading to the linear system in Eq. 2, when supplied with a *b* vector. These formalisms allow the definition of spaces to block through well-defined bounds for certain fluxes, bypassing the need for selecting specific EMs.

2 $$ \left[\begin{array}{ccc}{T}_{1,1}& \cdots & {T}_{1,n}\\ {}\vdots & \ddots & \vdots \\ {}{T}_{m,1}& \cdots & {T}_{m,n}\end{array}\right].v\ge \left[\begin{array}{c}{b}_1\\ {}\vdots \\ {}{b}_m\end{array}\right] $$

### Growth-coupled product synthesis

The objective of virtually all CSOMs is to find subnetworks in which the flux to be optimized is guaranteed to be active, considering some restrictions. Growth coupling with production occurs when there is at least one possible flux vector where the production flux *p* is kept above a threshold *p*_*min*_ and the growth pseudo-flux *b* is kept above a minimum *b*_*min*_. This definition is broad, and thus, two types of growth coupling can also be distinguished. It should also be noted that the original definition involves product and growth per substrate yields. Since our experimental conditions assume a fixed substrate uptake rate, these definitions have been adapted for flux values only.

Strong coupling occurs if **all** admissible flux vectors force *p* to be kept above *p*_*min*_ and at least one of these allows *b* to be kept above *b*_*min*_, implying there is always product synthesis and that its flux can be coupled with growth.

Weak coupling, on the other hand, imposes that all flux vectors where *b > b*_*min*_ must also force *p > p*_*min*_. This is a less demanding constraint since the product is not essential until the growth rate rises above a certain threshold.

For this work, we set the minimum product and growth thresholds for coupling at a very small value ε = 10^− 4^ to avoid arbitrarily discarding growth-coupled solutions with low production fluxes.

## Strain optimization algorithms

### Multi-objective evolutionary algorithms

In this study, we employ the OptGene method using the Strength Pareto Evolutionary Algorithm 2 (SPEA2) as representative of SB CSOMs. This approach allows for the definition of multiple objective functions [16], providing a flexible framework for defining appropriate ME goals. Unlike typical evolutionary algorithms where an aggregate function must be defined if multiple objectives are required, SPEA2 evaluates these separately and prioritizes those with the highest Pareto optimality.

The overall algorithm employed in this setup includes the following steps:

1. Generate an initial random set (population) of candidate strategies (individuals) containing reaction knockouts
2. Decode each individual and convert reaction deletions into flux constraints to be applied to the CB model
3. Determine the fitness value for each individual using a suitable objective function. This function can be linear or nonlinear.
4. Select the best individuals according to their fitness values. The assumption here is that fitter individuals will give rise to better offspring strategies.
5. Replace the population for a new one by applying mutation and crossover operators to the previous set of strategies, thus yielding new strategies that combine reaction knockouts from the fittest individuals.
6. Obtain the design strategies from the population if the stopping criteria are met. Otherwise, return to step 2.

To provide a better comparison with EMA-based CSOMs, two sets of fitness functions are used. The first is a typical approach in ME problems, maximizing both product and biomass fluxes, while the second represents a search for more robust strategies, enforcing growth-coupled compound synthesis at lower growth rates to reach growth-coupling phenotypes similar to those found with EMA-based CSOMs. Both are represented on Table 1 and described in the following.

**Table 1:** Overview of the evolutionary algorithm formulations used in this work. Both objective functions are represented, as well as other constraints that condition the acceptance of a given candidate as a valid solution

Formulation	Objective function #1	Objective function #2	Additional constraints
*EAw*	Z = *Max* (*v*_*b*_)	*Max*(*v*_*p*_)	None
*EAm*		*Max*(*W* = *Min* (*v*_*p*_))*s*. *t*. *v*_*b*_ = *Z*	Accept solution if ${v}_p>0\ if\ {v}_b>\frac{Z}{2}$

#### Biomass-product coupling maximization (*EAw*)

A multi-objective approach using maximum cell growth and production flux at maximum cell growth as two separate objective functions. This represents growth coupling as the strategies are evolved towards increased product and biomass production values but does not guarantee strong coupling as it inherently assumes biomass maximization.

#### Product minimum maximization (*EAm*)

This approach includes the maximum cell growth rate and minimum product flux at maximum growth as objectives. The latter is determined through FVA [41] by setting biomass production at its maximum and minimizing the product flux. Additionally, the cell must always synthesize the product (product flux above 0) if the growth rate is at 50% of the theoretical maximum value.

For this work, candidate strategies were subject to a maximum of 20 deletions. Although that amount of deletions is not acceptable for in vivo implementation, we have decided not to restrict the enumeration with a smaller strategy size to avoid discarding potentially useful phenotypes for our analysis. Since this is an evolutionary algorithm, a pool of solutions is evolved until a stopping criterion is reached, in this case being a cap of 10^5^ objective function evaluations. Due to the heuristic nature of the algorithm, it was executed 10 times for each case.

### MCSEnumerator

The MCSEnumerator approach is based on the findings of Ballerstein et al. [23] reporting the properties of dual networks based on original stoichiometric metabolic networks. For this problem, we first assume a *m*-by-*n* stoichiometric matrix **S** with reversible and irreversible reactions identified by sets of indices *r* and *i,* respectively.

The dual network/system is essentially a transposed and extended network based on the original, where reactions become metabolites and vice-versa and is represented in Eq. 3. The metabolites representing the original reactions are produced by newly added reactions (represented by vectors *v* and *h*) and consumed by the reactions added as part of the target space **T**. EFMs enumerated in this network in which fluxes in *w* are active are MCSs of the original network. The original mapping is obtained by checking which fluxes in the vectors *v* and *h* are active, and thus, satisfying the demand caused by the fluxes in *w*.

3 $$ {\displaystyle \begin{array}{l}\left[\begin{array}{cccc}{S}_i& I& -I& {T}_i\\ {}{S}_r& I& -I& {T}_r\end{array}\right].\left[\begin{array}{c}u\\ {}v\\ {}h\\ {}w\end{array}\right]\begin{array}{c}\ge 0\\ {}=0\end{array}\\ {}b.{w}^{\hbox{'}}>c;c>0\\ {}u\in {\mathrm{\Re}}^m\\ {}v,h,w\in {\mathrm{\Re}}_{0+}^n\end{array}} $$

In MCSEnumerator, this dual network is used as input for the k-shortest algorithm which is integrated in the linear problem by adding binary variables for each flux in *v* and *h* which are set to 1 if the respective flux is active or 0 otherwise. Additional constraints ensure that the split reaction pairs cannot be simultaneously active. Finally, the objective function is set towards the minimization of the number of active reactions in *v* and *h*.

The intervention problem framework described in the previous section is used to formulate cMCS enumeration problems, with all formulations written in the form **I**(**T, D**), with **T** and **D** representing flux spaces to block and keep. The desired space **D** can be defined as appropriate environmental conditions supplied as constraints. Additionally, a lower bound constraint on the biomass pseudo-reaction discards lethal cMCSs. Both **T** and **D** are matrices representing the linear coefficients for inequalities in the form **T.**v ≤ b, assuming *v* as the flux vector and *b* as a vector of size *c* with one value for each constraint to add.

#### Defining target spaces

Different combinations of constraints for the definition of the target flux space **T** were tested with MCSEnumerator. Four main constraint types can be summarized, namely:

1. **Product constraint**, indicating that the product must stay above a certain threshold. Product/Substrate and Product/Biomass yields, as well as simple product flux constraints were tested.
2. **Substrate constraint**, either fixed or with an upper bound on the maximum uptake rate. Can be considered as an environmental condition restricting the feasible solution space.
3. **Coupling constraint**, referring to additional flux bounds introducing assumptions to the problem to further restrict the solution space. In our setup, we have tested a constraint to set the flux of the maintenance ATP pseudo reaction.
4. **Biomass constraint**, where a biomass lower bound is set to further restrict the target flux space.

From this early analysis, we have determined that biomass constraints together with product yield constraints negatively affect computational speed, and do not result in additional solutions. Furthermore, we have also determined that non-fixed substrate constraints require an additional coupling component, otherwise leading to an infeasible enumeration problem. In such scenarios, it is possible that the origin point of the flux cone is included in the undesired space, for which there are no MCSs that can block it, should the problem only contain homogeneous constraints.

Three formulations were selected to consider different strain design objectives. Table 2 highlights the constraints used in these formulations, which are:

- ***MCSe***: The undesired space contains low product yield flux vectors with a maximum substrate uptake rate of *s*_*max*_ and maintenance ATP rate above *m*. A similar formulation is employed in *Escherichia coli* ME case studies with MCSEnumerator [22].
- ***MCSf***: Low product yield phenotypes are blocked, but assuming a fixed substrate uptake rate of *s*_*max*_. The aim is to eliminate the maintenance ATP assumption, while maintaining the origin point of the flux cone out of the undesired space.
- ***MCSw***: This formulation attempts to find less robust solutions by blocking low product flux phenotypes only when biomass fluxes are above a fraction F of *b*_*max*_ (maximum growth rate). The aim is to try and reach strategies with less strict demands regarding product synthesis and its coupling with growth. While many strategies may not allow growth-coupling at all, some weak-coupled cMCSs can theoretically be found using this formulation.

**Table 2:** Overview of the constraints for the undesired space (T) used in formulations involving enumeration of constrained minimal cut sets

Formulation	Product constraint	Substrate constraint	Coupling constraint	Biomass constraint
*MCSe*	$\frac{v_p}{v_s}\le {y}_{min}$	*v*_*s*_ ≤ *s*_*max*_	*v*_*atp*_ ≥ *m*	None
*MCSf*		*v*_*s*_ = *s*_*max*_	None	
*MCSw*	*v*_*p*_ ≤ *ε*	*v*_*s*_ = *s*_*max*_	None	*v*_*b*_ ≥ *F*. *b*_*max*_

## Filtering

Strategies from the optimization algorithms were filtered according to their expected growth-coupling strength. A filtering pipeline using FVA [41] was developed to ensure that three key conditions were met, namely:

- **Compliance with environmental conditions**: Strategies must be feasible with substrate uptake and maintenance ATP constraints, among others;
- **Growth rate**: Maximum mutant growth rates must reach at least 1% of the wild-type strain;
- **Growth-coupling with product**: Mutants must carry non-zero product flux with the cell at or above 90% of the maximum mutant growth rate (growth coupling phenotypes).

It is worth noting that while the *MCSe* and *MCSf* formulations would always lead to growth-coupled cMCSs, this is not the case for *MCSw* nor solutions based on evolutionary algorithms. Thus, this step is required to discard any strategy that does not lead to growth-coupled production.

## Analysis

A global assessment of the selected solution sets was performed regarding four key aspects:

- **Performance** – Predict production titres and robustness;
- **Pathways** – Compare overall pathway usage across strategy groups;
- **Phenotype** – Investigate individual strategies from various groups to find mechanisms leading to growth-coupling phenotypes;
- **Structure** – Assess the impact of individual knockouts in each strategy group relative to their phenotype/pathway pattern.

The constraints and objective functions used to obtain the productivity metrics are highlighted on Table 3. These metrics are:

- **Production robustness**: Minimum feasible production rate given a lower limit for cell growth. Production is considered robust for a certain biomass threshold, if the corresponding minimum rate is not null.
- **Predicted fluxes**: Assuming the pFBA flux distribution as the predicted phenotype, maximum cell growth, substrate uptake and production rate fluxes are obtained and considered as predictions of in vivo behaviour.
- **Production yield**: Biomass-product coupled yield [14] was used to assess the productivity of the mutant strains, since it takes cell growth, production rate and substrate uptake into account.

**Table 3:** Phenotype prediction methods used to obtain the performance metrics featured in this work

Method	Objective function	Constraints	Productivity metrics
pFBA	Min (|v|) subject to Max (v_biomass_)	Environmental conditions	Maximum cell growth = v_biomass_
			Production rate = v_product_
			Biomass-product coupled yield: $BPCY(v)=\frac{v_{biomass}\ast {v}_{product}}{v_{substrate}}$
FVA	Min (v_product_)	Environmental conditions v_biomass_ ≥ *x Max* (v_biomass_)	Production robustness at *x* % growth = *Min* (v_product_)

Using the pFBA flux distributions for all the mutant strains, it is possible to compare pathway usage across various sets of solutions. With the wild-type distribution as reference, the purpose is to find how different groups of strategies influence pathway usage.

Consider a *n*-by-*m* binary matrix *p* with *n* pathways and *m* reactions. Each element *p*_*nm*_ has a value of 1 if reaction *m* is part of a pathway *n*, and 0 otherwise. For each strategy with a flux distribution *v*, a “pathway distribution” is given by *d (v, p)*, a vector with *n* elements, one for each pathway, containing the number of active reactions for each pathway.

4 $$ d\left(v,p\right)=\frac{\sum_{i=1}^m\left({p}_{ji}.\left|\mathit{\operatorname{sgn}}\left({v}_i\right)\right|\right)}{\sum_{i=1}^m{p}_{ji}}\forall \mathrm{j}\in \left\{1,\dots, \mathrm{n}\right\} $$

For each strategy in a given group, a pathway distribution is generated according to the formula in Eq. 4, assuming *sgn(x)* as the sign function of *x*. Assuming this is performed for *s* solutions, a *s* × *n* matrix **P** can be assembled, containing all of the pathway distributions for the strategy set. The global difference from the wild-type can be found through determination of the mean value for each pathway in **P** and subtracting it to the corresponding value in the wild-type.

### Software

Enumeration of cMCSs was performed using *mcslibrary* [44], an open-source *Java* library implementing the methods described by Von Kamp and associates used for MCSEnumerator [22]. The tool, developed in-house, has been validated using the case studies that were also applied to MCSEnumerator. The analysis pipeline for the entire set of design strategies was also performed using this tool. Source-code and test scripts are available from the Git repository at https://github.com/MEWorkbench/mcslibrary. A graphical user interface implementing some functionalities of this library is available as a plugin (*optflux-mcs*) for the OptFlux ME software platform [45]. Currently on its third major release, it is an open-source tool that allows users to load metabolic models and use a wide array of phenotype prediction methods and CSOMs in a user-friendly setting.

Our SPEA2 workflow is currently available as part of the MEWorkbench’s *mewcore* library containing core methods used within OptFlux for simulation, analysis and strain optimization. Source-code is available on the Git repository at https://github.com/MEWorkbench/mewcore. The evolutionary algorithm is part of JECoLi (Java Evolutionary Computation Library), a library implementing generic evolutionary algorithms with source code available at https://github.com/jecoli/jecoli.

Data analysis and plots shown in this work were processed using the R programming language. Scripts to perform this analysis are available at http://www.bio.di.uminho.pt/csomcomparison/.

## Availability and requirements

**Project name:** mcslibrary.

**Project home page:** https://github.com/MEWorkbench/mcslibrary

**Operating systems(s):** Platform independent.

**Programming language:** Java.

**Other requirements:** IBM® ILOG® *CPLEX*® Optimizer version 12 or higher, Java® 1.7 or higher.

**License:** GNU Lesser General Public License 2.1.

## Additional file

Additional file 1: Compound overproduction in *Escherichia coli* using minimal cut set based strain design. (DOCX 296 kb)

## Data Availability

The datasets generated and analysed during the current study are available at http://www.bio.di.uminho.pt/csomcomparison/.

## Abbreviations

ACK: Acetate kinase
ACOAH: Acetyl-CoA hydrolase
ALDD: Aldehyde dehydrogenase
ATP: Adenosine triphosphate
BPCY: Biomass-product coupled yield
CB: Constraint-based
cMCS: Constrained minimal cut set
CSOM: Computational strain optimization method
EA: Evolutionary algorithm
EM: Elementary mode
EMA: Elementary modes analysis
FBA: Flux Balance Analysis
FVA: Flux Variability Analysis
G6PDH: Glucose 6-phosphate dehydrogenase
GABA: Gamma-aminobutyric acid
GAPD: Glyceraldehyde-3-phosphate dehydrogenase
GSMM: Genome-scale metabolic model
IDH: Isocitrate dehydrogenase
KEGG: Kyoto Encyclopedia of Genes and Genomes
MCS: Minimal cut set
ME: Metabolic engineering
MILP: Mixed-integer linear programming
MIP: Mixed-integer programming
NADP: Nicotinamide adenine dinucleotide phosphate
NADP(H): Nicotinamide adenine dinucleotide phosphate (reduced)
pFBA: Parsimonious Flux Balance Analysis
PGCD: Phosphoglycerate dehydrogenase
PGI: Glucose-6-phosphate isomerase
PGK: Phosphoglycerate kinase
POX: Pyruvate oxidase
PPP: Pentose phosphate pathway
PTA: Phosphate acetyltransferase
PYRDC: Pyruvate decarboxylase
SB: Simulation-based
SDH (1/2): Succinate dehydrogenase (subunit 1/2)
SPEA: Strength Pareto Evolutionary Algorithm
TCA: Tricarboxylic acid

## Footnotes

1
https://github.com/MEWorkbench
