# Supplementary material for: Comparison of pathway analysis and constraint-based methods for cell factory design
Source: BMC Bioinformatics. 2019 Jun 20;20:350. doi: 10.1186/s12859-019-2934-y (PMC6585037; doi:10.1186/s12859-019-2934-y)
Supplement: Supplementary file 1 — Compound overproduction in Escherichia coli using minimal cut set based strain design. (DOCX 296 kb) [file 12859_2019_2934_MOESM1_ESM.docx]

## Compound overproduction in *Escherichia coli* using minimal cut set based strain design

With the intent of assessing the performance of the new formulations proposed in this work, compared to previous MCS-based design strategies, two recent case studies involving compound overproduction with *Escherichia coli*, namely anaerobic ethanol production and aerobic itaconic acid production, were considered.

The first case study was presented by Von Kamp and co-workers along with MCSenumerator [1] and was tested on the *i*AF1260 GSMM for *Escherichia coli* containing 2382 reactions [2]. Using MCSs, the authors intended to enable ethanol overproduction at high product per substrate yields, for which two constraints were tested for both product yield demands and substrate uptake. The resulting MCSs could grant the desired product yields with a relatively low number of modifications.

The second case study is featured in a recent work by Harder and colleagues aiming at overproducing itaconic acid in aerobic conditions under glucose [3]. In this work, a central carbon metabolism model of *Escherichia coli* was used including modifications representing the *in vivo* strain to which the intervention strategies were applied.

In the aforementioned studies, cMCSs were determined using a formulation identical to the MCSe formulation that was previously described.

Since both case studies already use models and environmental conditions that allow weakly growth-coupled compound production by default, the MCSw formulation was discarded.

### Anaerobic ethanol production

In this case study, four different environmental and product demand constraints were tested. The results from the MCSe formulation match those found on the original publication, as expected, since the enumeration problem in this case is identical, providing a strategy set to compare with the newly proposed formulation. The results for this case study are highlighted on Table 1, Figure 5 and Figure 6.

Table 1 - Overview of the experimental setup and results for the anaerobic ethanol production case study. The minimum product yield and maximum glucose uptake fields represent the different constraints considered for the definition of the undesired space.

| **Formulation** | **Minimum product yield**  ([Product]. [Glucose]^-1^) | **Maximum glucose uptake**  (mmol. gDW^-1^.h^-1^) | **Number of cMCSs** |
| --- | --- | --- | --- |
| MCSe | 1.4 | 10.0 | 8342 |
|  |  | 18.5 | 8819 |
|  | 1.8 | 10.0 | 1987 |
|  |  | 18.5 | 4618 |
| MCSf | 1.4 | 10.0 | 384 |
|  |  | 18.5 | 2850 |
|  | 1.8 | 10.0 | 0 |
|  |  | 18.5 | 0 |

#### Productivity metrics

At a first glance, the MCSf formulation generally leads to less solutions. This can be explained by the larger size of the undesired solution space, in which the maintenance ATP flux is not fixed. Strategies from this formulation were not found for higher ethanol production yield demands.
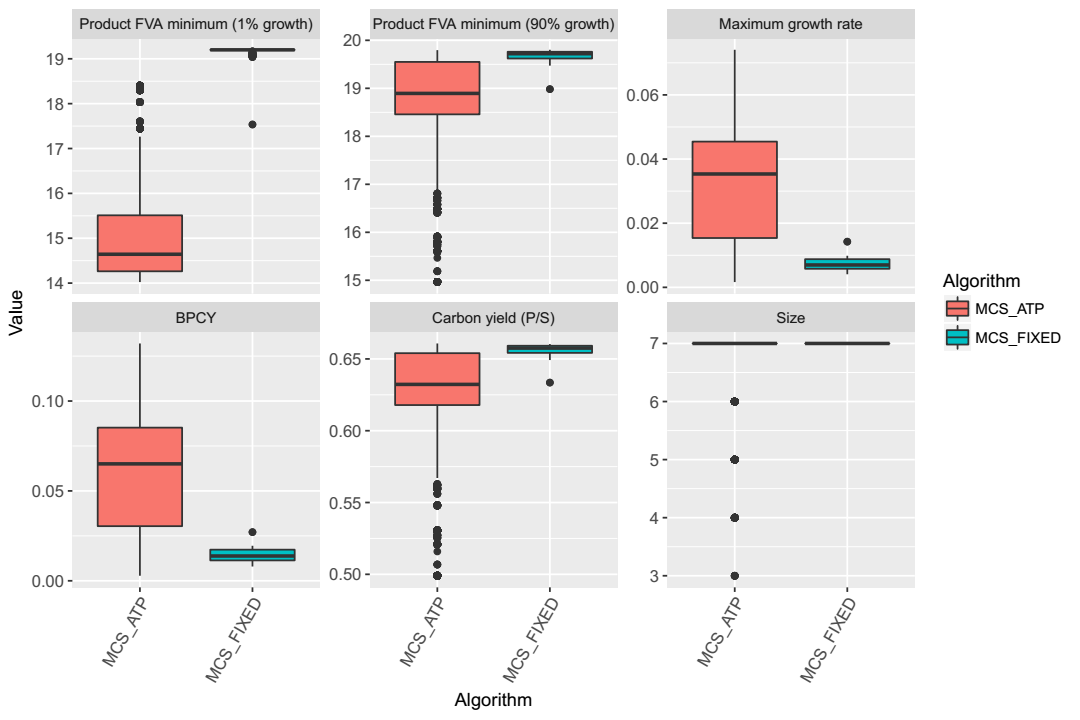


Figure 5 - Overview of productivity and robustness metrics from cMCSs for anaerobic ethanol production in *E. coli* with a minimum production yield of 1.4 mmol/gDW/h and maximum glucose uptake of 10 mmol/gDW/h. MCS_ATP and MCS_FIXED correspond, respectively to MCSe and MCSf formulations.

Additionally, the maintenance ATP constraint is essential to obtain strategies with higher growth-coupled yield demands. This is represented by the high BPCY and growth rates for strategies from the MCSe set. The MCSf formulation leads to higher production robustness, with virtually all strategies leading to higher production flux values at 1% of the mutant growth rate. The size of these strategies is considerably higher than those provided by the MCSe set, which could explain the lower growth rates.

#### Strategy structure


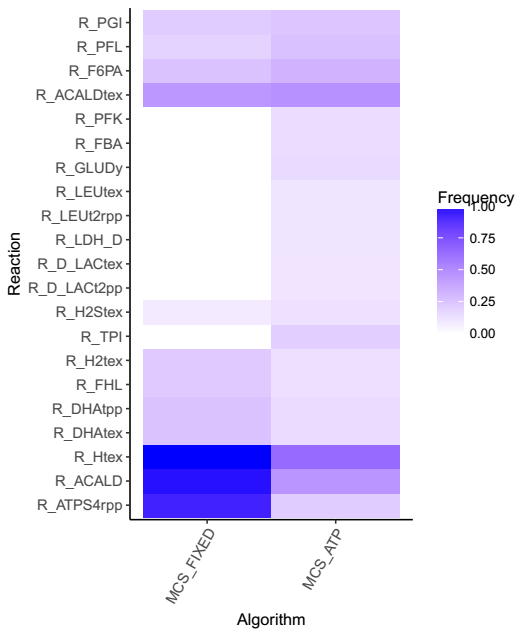


Figure 6 - Most common reaction knockouts present in cMCSs for strongly growth-coupled production of ethanol in anaerobic conditions. MCS_ATP and MCS_FIXED correspond, respectively to MCSe and MCSf formulations.

### Aerobic itaconic acid production

As already performed in the previous case study, MCSf and MCSe formulations were applied using the parameters described by the original authors. The biomass reaction and exchange fluxes were not allowed to be a part of any cMCSs. The MCSe and MCSf formulations yielded, respectively, 945 and 1034 cMCSs. Unlike the previous case study, a larger number of strategies up to size 9 were found using the newly proposed formulation.

#### Productivity metrics

The removal of the maintenance ATP constraint in the MCSf formulation led to a moderate increase in production robustness, as demonstrated on Figure 7. Both minimum production flux and carbon yields are increased, reaching almost 85%. Despite this, the growth/robustness trade-off is still present as the maximum growth rates in MCSf solutions are decreased. Biomass-coupled product yields are generally lower, with the increase in production not being enough to compensate for the reduced growth rates. Strategy sizes are similar but distributed differently, as it can be observed that there is a greater number of strategies above size 6 and 7.


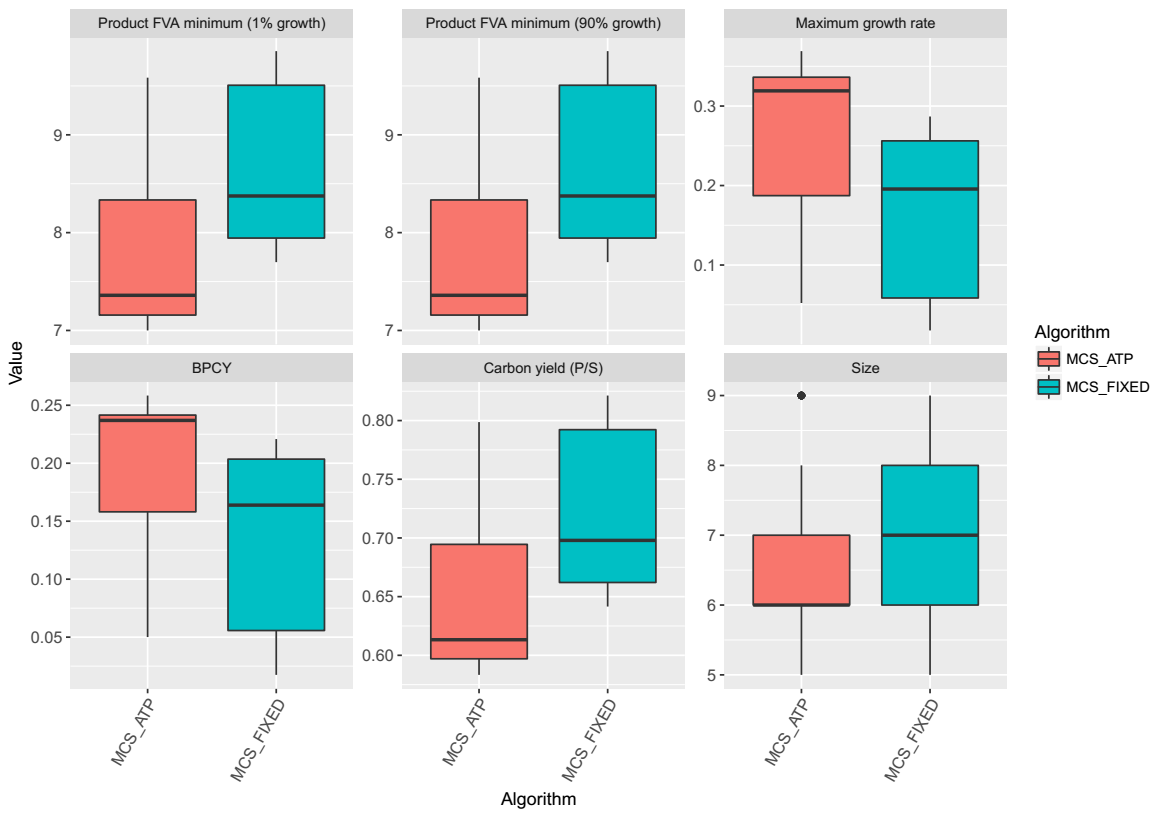


Figure 7 - Overview of productivity and robustness metrics from cMCSs for aerobic itaconic acid production. MCS_ATP and MCS_FIXED correspond, respectively to MCSe and MCSf formulations.

#### Strategy structure and phenotypes

The knockouts and resulting phenotypes from the various strategy sets were analysed, with key results shown on Figure 8. The most significant phenotypical differences between both solution groups can be identified at the TCA cycle and alternate carbon metabolism. In this model, itaconic acid is produced from cis-aconitate and, thus, citrate synthesis occurs in similar values for both groups.

MCSf solutions show higher usage of the glyoxylate shunt and methylglyoxal metabolism as an alternative for pyruvate synthesis (through dehydrogenation of lactate derived from methylglyoxal) and a concurrent decrease in pyruvate kinase flux. This is expected as the frequency of triose phosphate isomerase knockouts is greater, which forces dihydroxyacetone to be metabolized through pathways other than glycolysis. Acetate production is decreased in MCSf strategies, which is expected as there is an increased frequency in enzymes related to that pathway (ACK, POX, PTA) in this group.


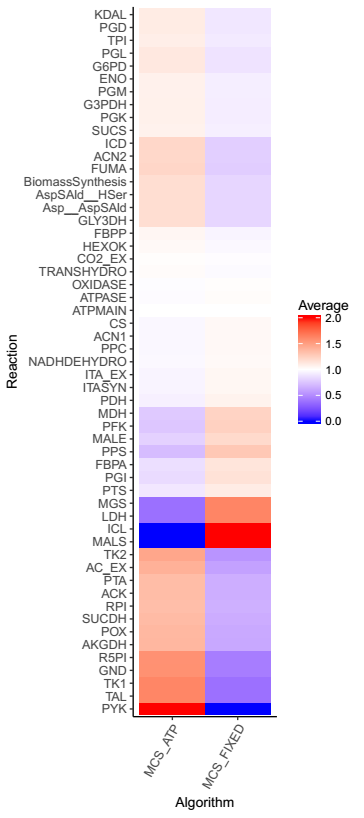

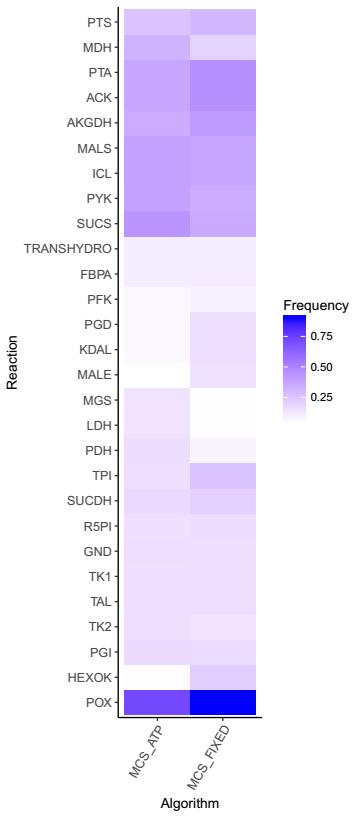


Figure 8 – **Left**: Most common knockouts and respective frequencies present in cMCSs for the itaconic acid production case study. **Right**: Average flux values for each solution group represented as percentages of the mean overall flux for both groups. Blue represents smaller proportions (lower than mean), while red represents higher proportions (above the mean). MCS_ATP and MCS_FIXED correspond, respectively to MCSe and MCSf formulations.

1. von Kamp A, Klamt S. Enumeration of Smallest Intervention Strategies in Genome-Scale Metabolic Networks. PLoS Comput Biol. 2014;10. doi:10.1371/journal.pcbi.1003378

2. Feist AM, Henry CS, Reed JL, Krummenacker M, Joyce AR, Karp PD, et al. A genome-scale metabolic reconstruction for Escherichia coli K-12 MG1655 that accounts for 1260 ORFs and thermodynamic information. Mol Syst Biol. 2007;3: 1–18. doi:10.1038/msb4100155

3. Harder B-J, Bettenbrock K, Klamt S. Model-based metabolic engineering enables high yield itaconic acid production by Escherichia coli. Metab Eng. Elsevier; 2016;38: 29–37. doi:10.1016/j.ymben.2016.05.008
